# Molecular Characterization Based on MLST and ECDC Typing Schemes and Antibiotic Resistance Analyses of *Treponema pallidum* subsp. *pallidum* in Xiamen, China

**DOI:** 10.3389/fcimb.2020.618747

**Published:** 2021-02-19

**Authors:** Dan Liu, Shu-Min He, Xiao-Zhen Zhu, Li-Li Liu, Li-Rong Lin, Jian-Jun Niu, Tian-Ci Yang

**Affiliations:** ^1^ Center of Clinical Laboratory, Zhongshan Hospital, School of Medicine, Xiamen University, Xiamen, China; ^2^ Institute of Infectious Disease, School of Medicine, Xiamen University, Xiamen, China

**Keywords:** syphilis, *Treponema pallidum* isolates, strain typing, ECDC, MLST, antibiotic resistance

## Abstract

In total, 49 clinical samples were analyzed using two typing schemes, Enhanced Centers for Disease Control and Prevention (ECDC) and multilocus sequence typing (MLST), to describe the molecular characteristics of circulating *Treponema pallidum* isolates in Xiamen between 2016 and 2017. In addition, genetic mutations potentially related to antibiotic resistance of *T. pallidum* were also analyzed. Forty five samples were fully typed by ECDC, and 14 different subtypes were detected. The most common subtype was 16d/f (24.4%), followed by 14d/f (20.0%). All forty nine samples were successfully typed by MLST, while only four allelic profiles were identified, including three SS14-like profiles and one Nichols-like profile. Among them, the major allelic profile was 1.1.8 (85.7%). Interestingly, the allelic profile 1.3.1 widespread in Europe and North America was not detected in this region. Additionally, A2058G mutation in 23S rRNA was found in all detectable samples (38/38), and no mutation in 16S rRNA was observed (36/36). Four non-synonymous single-nucleotide polymorphisms in penicillin-binding protein genes were found in the 35 samples eligible for Sanger sequencing. Among them, the variant in *tp0500* (P564I) can only be found in the SS14-like isolates. Homoplastic changes in *tp0760* (I415F/I415M) and *tp0705* (A506V/A506T) were found. Moreover, the variant *tp0705* A506V and the variant *tp0705* A506T separately appeared in the SS14-like isolates and Nichols-like isolates, respectively. This study showed that the genotypes of *T. pallidum* isolates in Xiamen between 2016 and 2017 were different from those in other geographic areas. The resistance-related variants of *T. pallidum* isolates identified in this study could provide awareness for clinicians in the treatment of syphilis.

## Introduction

Syphilis, caused by *Treponema pallidum* subsp. *pallidum*, is a chronic sexually transmitted disease. Recently, the number of cases of syphilis has dramatically increased in many countries, including China (Shen et al., 2018; Liu et al., 2019). Because of the difficulty in culture of *T. pallidum in vitro*, *T. pallidum* was cultured through *in vivo* rabbit testicular models in the past decades, which greatly hindered the understanding of *T. pallidum* biology (Ho and Lukehart, 2011). To date, molecular characterization has been an important tool to understand *T. pallidum* biology. Strain typing, as a powerful tool for determining the diversity of circulating *T. pallidum* isolates and the dynamic transmission of the infection, is widely applied in many countries (Peng et al., 2011; Dai et al., 2012; Gallo Vaulet et al., 2017; Giacani et al., 2018).

In 1998, the Centers for Disease Control and Prevention (CDC) firstly introduced a CDC typing scheme based on determining the number of 60-bp repeats in the *arp* gene and sequence differences by restriction fragment length polymorphism analysis (RFLP) in the *tpr* genes to distinguish among subtypes of *T. pallidum* (Pillay et al., 1998). Then, the scheme was supplemented by additional sequencing of a portion of the *tp0548* gene and designated as the enhanced CDC (ECDC) typing system (Marra et al., 2010). The two typing schemes were commonly used with over 3,000 clinical *T. pallidum* isolates worldwide, and they enabled identification of several associations between the subtypes and specific clinical status, including the 14d/f type with neurosyphilis and the 14i/a with the serofast status (Marra et al., 2010; Zhang et al., 2017). However, the typing scheme has weaknesses, including the low amplification efficiency of the *tpr* genes and possible instability of the *arp* and *tpr* loci (Mikalová et al., 2013). Therefore, sequencing-based molecular typing based on sequencing of the *tp0136*, *tp0548*, and 23S rRNA genes has been introduced in parallel (Flasarova et al., 2012). With the prevalence of SS14-like strains worldwide, one of the two main *T. pallidum* clades, sequencing-based molecular typing was supplemented with additional sequencing of the *tp0705* gene, known as the multilocus sequence typing (MLST) system, to greatly increase the genotype resolution power for SS14-like isolates (Grillova et al., 2018). The typing scheme based on direct sequencing of typing loci effectively improved the success rate of fully typed isolates and ensured stability. Moreover, it enabled the construction of phylogenetic trees to trace infections and distinguish *T. pallidum* from other treponemal subspecies (Grillova et al., 2018; Grillova et al., 2019a).

Additionally, there is no effective vaccine to prevent syphilis infection, antibiotic treatment remains an important strategy for syphilis control. However, the emergence of antibiotic-resistant *T. pallidum* isolates, similar to other bacteria that increase antimicrobial resistance due to the use of antibiotics, has always been a concern. Recently, a high prevalence of azithromycin resistance to *T. pallidum* isolates in geographically different areas in China and treatment failure cases have been reported (Zhou et al., 2010; Chen et al., 2013). It has been demonstrated that there is a strong association between the A2058G and A2059G mutations in 23S rRNA and macrolide resistance (Lukehart et al., 2004; Molini et al., 2016). Mutations in 16S rRNA was indicated to associate with tetracycline resistance in *T. pallidum* isolates (Wu et al., 2014). Moreover, previous analysis of *T. pallidum* genomes have shown amino acid changes in penicillin-binding proteins, although the clinical relevance of these changes is unclear so far (Pinto et al., 2016; Sun et al., 2016). This fact reminds us of the necessity to detect mutations in relevant genes and investigate the prevalence of resistances in a region.

Hence, we applied the two commonly used typing schemes, ECDC and MLST, to describe the circulating genotypes of *T. pallidum* isolates in Xiamen and comprehensively understand the epidemic change in *T. pallidum* between 2016 and 2017. In addition, we explored the 23S rRNA gene, 16S rRNA gene and three penicillin-binding protein (*pbp*) genes (*tp0500*, *tp0760*, and *tp0705*) for potential mutations associated with antibiotic resistance in the *T. pallidum* isolates.

## Materials and Methods

### Collection of Clinical Samples

Between 2016 and 2017, 78 clinical samples were collected from 78 patients with syphilis in Xiamen, China, including 57 lesion swabs and 21 cerebrospinal fluid (CSF) samples. According to the US Centers for Disease Control and Prevention (CDC) and the European CDC (ECDC) guidelines (Janier et al., 2014; Workowski and Bolan, 2015), syphilis patients were clinically diagnosed based on clinical findings with laboratory tests including serological tests and dark-filed microscopy. Serology tests were performed using the rapid plasma regain (RPR) test (InTec, Xiamen, China) and *Treponema pallidum* particle agglutination (TPPA) test (Fujirebio, Tokyo, Japan). Patients were considered to have syphilis when one of the tests (serology or/and dark-field microscopy) was positive. Clinical data of the enrolled patients were collected.

### Isolation and Detection of Treponemal DNA

DNA was isolated from the patient’s lesion swabs or CSF using a QIAamp DNA Mini Kit (Qiagen, Hilden, Germany) in accordance with the manufacturer’s protocol (Tong et al., 2017; Zhang et al., 2020). Real-time PCR targeting *tp0574* was performed to determine the copies of treponemal DNA. The DNA samples that tested positive were stored at -20°C for the next procedure.

### Molecular Typing of *Treponema pallidum* Isolates

The ECDC typing scheme, including the determination of the number of 60-bp repetitions in the *arp* genes, difference analysis in RFLP of the *tprE*, *G*, and *J* genes, and sequencing of an 83-bp region in the *tp0548* gene, were performed as described previously (Marra et al., 2010). MLST was performed as described previously (Grillova et al., 2018). Briefly, three loci, *tp0136*, *tp0548*, and *tp0705*, were amplified, and the purified amplicons were sequenced. A high-fidelity PCR polymerase, KOD FX Neo polymerase (TOYOBO, Osaka, Japan), was used for amplification of the two typing schemes. DNA sequencing was performed by Sangon Biotech Company (Shanghai, China).

### Detection of Mutations Potentially Associated With Antibiotic Resistance

The 23S rDNA and 16S rDNA genes were amplified and sequenced following previously described methods (Xiao et al., 2016). Three *pbp* genes, *tp0500*, *tp0760*, and *tp0705*, were amplified and sequenced to identify the reported mutations and the development of new mutations (Sun et al., 2016).

### Phylogenetic Analyses

Sequence Matrix 1.8 software was used for sequence concatenation. Phylogenetic trees were generated with MEGA 5 using the Tamura 3-parameter model and 1,000 pseudorandom bootstrap replicates.

## Results

### Clinical Characteristics of Syphilis Patients

**Figure 1:**
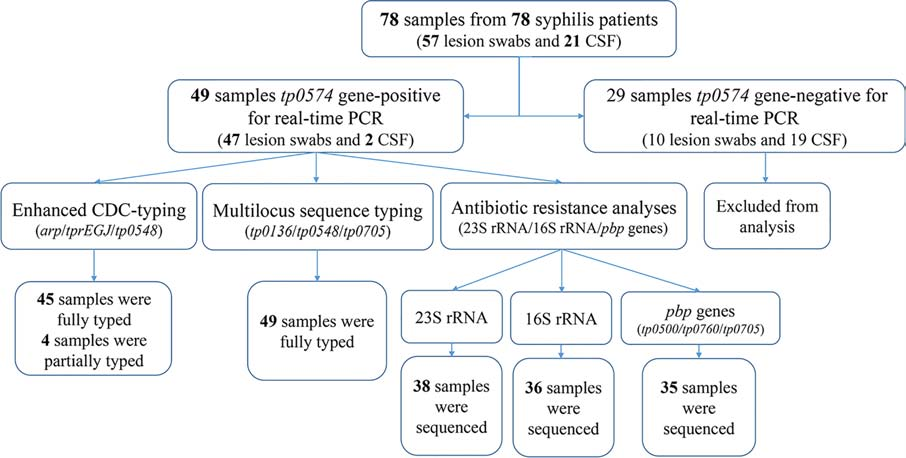
Flow chart of patients and samples included in the study.

Treponemal DNA was detected positively in 49 syphilis patients, including 29 primary syphilis patients and 18 secondary syphilis patients (lesion swabs collected) and two tertiary syphilis patients (CSF collected) (**Figure 1**). The samples from the 49 syphilis patients were further analyzed using ECDC and MLST. The clinical information for the 49 syphilis patients is listed in **Table 1**. Most of the patients included in this study were male (83.7%), with a mean age of 46 years. Regarding diagnostics and clinical status, the majority of recruited patients (95.9%) were at an early syphilis stage, and 59.2% were patients with primary syphilis. Out of 49 samples, for which dark field microscopy was performed, 83.7% were positive, showing an appreciable microscopy-positive rate. Except for three primary syphilis patients with negative rapid plasma regain (RPR), all other patients were positive for serological tests, including RPR and *T. pallidum* particle agglutination (TPPA).

**Table 1:** Clinical characteristics of 49 syphilis patients.

Characteristics	Patients (n=49)
Gender (n, %)	
Male	41 (83.7%)
Female	8 (16.3%)
Age (median, IQR)	
Male	46 (32–62)
Female	40 (27–47)
Diagnostic (symptomatic, n, %)	
Genital ulcer	25 (51.0%)
Condyloma	14 (28.6%)
Rash	5 (10.2%)
Lymphadenopathy	3 (6.1%)
Neurologic symptoms	2 (4.1%)
Clinical phase (n, %)	
Primary syphilis	29 (59.2%)
Secondary syphilis	18 (36.7%)
tertiary syphilis	2 (4.1%)
Dark-field microscopy (n, %)	
Positive	41 (83.7%)
Negative	8 (16.3%)
Serum RPR titer (Median, IQR)	
Primary	1:16 (1:2–1:32)
Secondary	1:16 (1:4–1:32)
Tertiary^a^	–
Serum TPPA (Median, IQR)	
Primary	1:320 (1:160–1:1,280)
Secondary	1:1280 (1:320–1:2,056)
Tertiary^a^	–

IQR, interquartile range; RPR, rapid plasma regain; TPPA, T. pallidum particle agglutination.

^a^The number of samples collected from tertiary syphilis patients were only two. The serum RPR titer was 1:8 and 1:16, respectively. And the serum TPPA titer was 1:5,120 and 1:10,240.

### Typing of Clinical Samples Based on ECDC Typing Scheme

**Figure 2:**
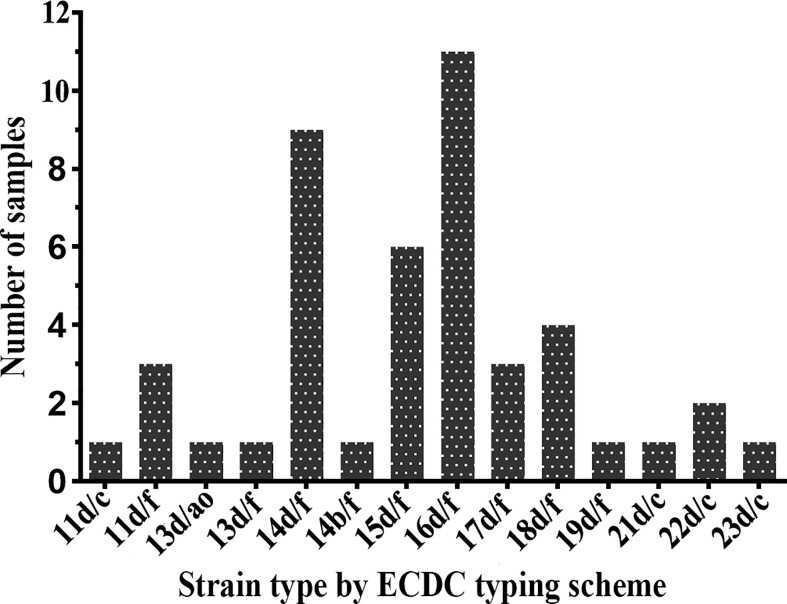
Distribution of *Treponema pallidum* subtypes by Enhanced Centers for Disease Control and Prevention (ECDC) typing scheme between 2016 and 2017.

Forty-five samples (91.8%) were fully typeable with the ECDC typing system (including the *arp*, *tprE/G/J*, and *tp0548* loci), and four samples were partially typed. The plurality of the fully typeable specimens belonged to strain type 16d/f, accounting for 24.4% (11/45). Strain type 14d/f was found to be the second most common strain, accounting for 20.0% (9/45), followed by 15d/f (13.3%), 18d/f (8.9%), 11d/f (6.7%), 17d/f (6.7%), and 22d/c (4.4%). All other subtypes, including 11d/c, 13d/**ao**, 13d/f, 14b/f, 19d/f, 21d/c, and 23d/c, were detected only once (**Figure 2**). In terms of the three typing loci, a total of 11 *arp* types (11, 13, 14, 15, 16, 17, 18, 19, 21, 22, and 23) were identified in these specimens, of which type 16 was the most commonly detected (11/45, 24.4%), followed by type 14, accounting for 22.2% (10/45). Two *tpr* RFLP patterns (b and d) were found, the majority of which belonged to type d. For the *tp0548* locus, three sequence-types (c, f, and **ao**), including a new type **ao**, were identified.

### Typing of Clinical Samples Based on MLST Typing Scheme

**Figure 3:**
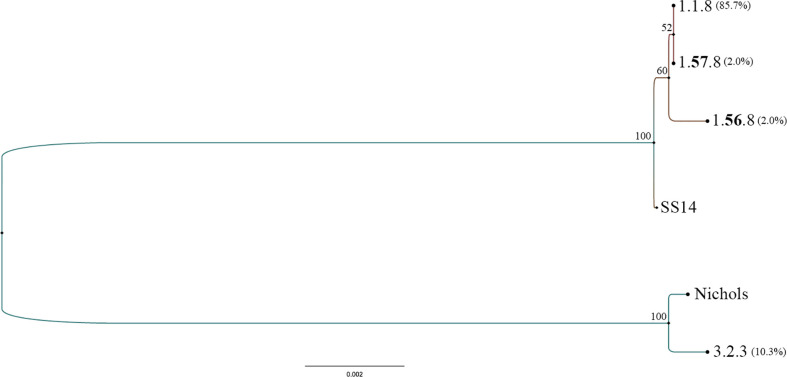
Phylogeny of allelic profiles obtained from all typed samples from 49 syphilis patients by multilocus sequence typing (MLST) typing scheme. *Treponema pallidum* Nichols (CP004010.1) and *T. pallidum* SS14 (CP04011.2) concatenated sequences of typing loci were used as reference.

**Figure 4:**
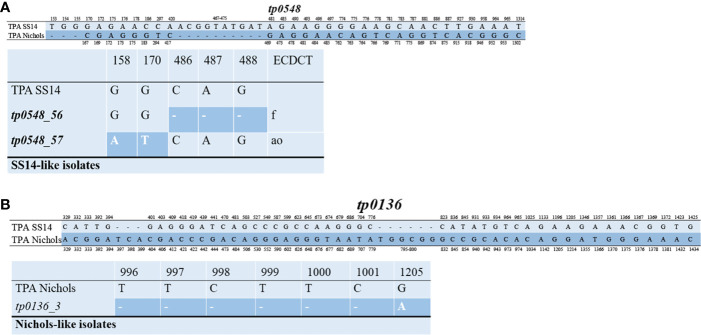
Alignment of the sequences in the *tp0548* and *tp0136* locus. Only position containing nucleotide variants are shown. Deletions are shown with dashes. **(A)** Upper part represents nucleotide difference between reference TPA SS14 (CP004011.1) and TPA Nichols (CP004010.2) in the *tp0548* locus. Lower patter represents the novel sequence identified in SS14-like isolates in the study using MLST (coordinates correspond to TPASS_0548) and the translation to ECDCT subtypes is shown in the last column. **(B)** Upper part represents nucleotide difference between reference TPA SS14 (CP004011.1) and TPA Nichols (CP004010.2) in the *tp0136* locus. Lower patter represents the sequence identified in Nichols-like isolates in the study by MLST (coordinates correspond to TPANIC_0136).

The 49 samples were fully typed at the *tp0136*, *tp0548*, and *tp0705* loci by the MLST typing system. In contrast to the ECDC typing system results, only four different allelic profiles (including 1.1.8, 1.**56**.8, 1.**57**.8, and 3.2.3) were observed for *T. pallidum* isolates circulating in Xiamen (**Figure 3**). Among them, the 1.1.8 allelic profile (the SS14-like profile) was the most common (85.7%, 42/49). Two additional new allelic profiles (1.**56**.8 and 1.**57**.8) belonging to the SS14-like group contained novel variants in the *tp0548* locus (**Figure 4A**). In addition, we found one profile that belonged to the Nichols-like group (3.2.3). Notably, the *tp0136* locus of the strains belonging to Nichols-like group were all contained, apart from one single-nucleotide variant (SNV) (G1205A), a 6-nucleotide deletion (TTCTTC) related to TPANIC_0136 (GenBank accession number CP004010.2) (**Figure 4B**). Then, we compared the discrimination ability of the two typing schemes for these 49 samples and found that the common allelic profile 1.1.8 was further divided into nine subtypes by ECDC. However, the most prevalent subtypes, 16d/f and 14d/f, were not further divided by MLST (**Table 2**).

**Table 2:** Comparison of fully typed samples using Enhanced Centers for Disease Control and Prevention typing (ECDCT) and multilocus sequence typing (MLST). Only samples with fully typed in both typing schemes are shown.

_Allelic profile_^Subtypes^	_11d/c_	_11d/f_	_13d/f_	_13d/ao_	_14b/f_	_14d/f_	_15d/f_	_16d/f_	_17d/f_	_18d/f_	_19d/f_	_21d/c_	_22d/c_	_23d/c_
1.1.8		3	1		1	9	6	11	3	3	1			
1.**56**.8				1										
1.**57**.8										1				
3.2.3	1											1	2	1

The bold value indicates the newly identified alleles of tp0548.

### Prevalence of Potential Mutations Associated With Antibiotic Resistance in the *Treponema pallidum* Isolates

The 23S rRNA gene locus was amplified in 38 samples (out of 49) and revealed by Sanger sequencing. No isolates were found to harbor the A2059G mutation; instead, all of them harbored the A2058G mutation, which meant that the isolates belonging to the SS14-like group or Nichols-like group were potentially resistant to macrolides (**Table 3**). Additionally, the sequences of the 16S rRNA gene locus, potentially associated with tetracycline resistance, from 36 samples were obtained, and no mutations were found. Additionally, we detected mutations in the genes encoding penicillin-binding protein of *T. pallidum* (*tp0500*, *tp0760*, and *tp0705*). A total of 35 samples were fully identified, and four non-synonymous single-nucleotide polymorphisms (SNPs) were found. A variant in *tp0500* (P564I) was exclusively present in the isolates belonging to the SS14-like group. And two SNPs affecting the same amino acid position in *tp0760* (I415F/I415M) and *tp0705* (A506V/A506T) were identified. In *tp0760*, the variant *tp0760* I415F was more frequently detected. In *tp0705*, the variant *tp0705* A506V only appeared in the SS14-like isolates and that the variant *tp0705* A506T only appeared in the Nichols-like isolates. In addition, we found a new non-synonymous SNP in the position 1,360 (C->T) of *tp0705* (P454S) in the X-12 isolate, which is beyond the sequence used for the MLST analysis (**Table 3**).

**Table 3:** Potential mutations associating with antibiotic resistance in *Treponema pallidum* isolates.

Allelic profile	23S rRNA	16S rRNA	*pbps*			Genetic group
			*tp0500*	*tp0760*	*tp0705*	
1.1.8	A2058G	Wild-type	C1691T (P564L)	A1243T (I415F) or C1245G (I415M) or Wild-type	C1517T (A506V) C1360T* (P454S)	SS14-like
1.56.8	A2058G	Wild-type	C1691T (P564L)	Wild-type	C1517T (A506V)	SS14-like
1.57.8	A2058G	Wild-type	C1691T (P564L)	A1243T (I415F)	C1517T (A506V)	SS14-like
3.2.3	A2058G	Wild-type	Wild-type	Wild-type	G1516A (A506T)	Nichols-like

*a new non-synonymous SNP in tp0705 (P454S) was found in the X-12 isolate.

## Discussion

In this study, we firstly explored the characteristics of *T. pallidum* isolates circulating in Xiamen, China. Two typing schemes (ECDC and MLST) were applied to determine the diversity of the *T. pallidum* isolates. In addition, we surveilled 23S rRNA mutations and 16S rRNA mutations to track the distribution of resistant strains in Xiamen. And we detected the genes encoding penicillin-binding proteins to monitor the reported mutations and identify the development of new mutations, although phenotypic or clinical penicillin resistance has never been documented in more than 60 years of clinical treatment for syphilis.

The study included 49 samples from 49 syphilis patients, of which 45 samples were fully characterized using the ECDC typing scheme. The results showed that the most common genotype in Xiamen was 16d/f (24.4%), while the prevalent genotype (14d/f) in Shanghai, Jiangsu, and Hunan (Dai et al., 2012; Xiao et al., 2016; Zhang et al., 2017) was the secondary, although the proportion of the two genotypes was basically equal (24.4 *vs*. 20.0%). This result suggested that there was a difference in the geographic distribution of syphilis-causing isolates. Moreover, the strain distribution evolved over time (Giacani et al., 2018), as previously described, with the introduction and loss of some strains in Seattle from 1999 to 2008 and the replacement of the prevalent subtype (14d/g replacing 14d/f) (Marra et al., 2010). Continuous tracking of the distribution of strains in Xiamen should be encouraged to determine whether the subtype 16d/f completely replace the 14d/f or whether a new strain type will appear and become prevalent.

In this study, all the samples were fully typed using a recently proposed typing scheme, MLST, which effectively avoids the difficulty in amplifying and analyzing tandem repeats in the *arp* and ambiguous restriction patterns in the *tprE/G/J* genes. As revealed in this study, only four allelic profiles were found in Xiamen, demonstrating less genetic diversity in comparison with that in other countries where MLST has been performed (Grillova et al., 2018; Pospisilova et al., 2018; Grillova et al., 2019b; Marangoni et al., 2019). Moreover, the most common allelic profile (1.1.8) identified in Xiamen between 2016 and 2017 was less common in other countries, and the most common allelic profile (1.3.1) reported in other areas was not found in this region. This result might indicate that the most prevalent allelic profile across the globe has not yet been introduced into China and that the strains tend to remain restricted to specific geographic areas. Notably, our study only provided a snapshot of *T. pallidum* isolates circulating in one area of China (Xiamen), and more research using MLST is needed to reveal the diversity of *T. pallidum* isolates in different areas of China to track the transmission of syphilis. In summary, we applied two typing schemes to investigate the diversity of *T. pallidum* isolates in Xiamen. The common allelic profile 1.1.8 was further divided by ECDC; however, the most prevalent subtype by ECDC was not further divided by MLST, which might be a consequence of less genetic diversity in the typing loci. It also indicated that the ECDC typing scheme might be better than MLST to investigate the genetic diversity of *T. pallidum* isolates in Xiamen. Of course, this study also demonstrated that the two typing schemes were independent and would be useful in different aspects to explore the epidemiology and evolution of *T. pallidum* isolates. In addition, MLST based on sequencing data yielded phylogenetic trees to better understand the epidemiology of the two genetically distinct groups of *T. pallidum* isolates. As described in previous studies (Beale et al., 2019), most of the sequence-characterized clinical *T. pallidum* isolates belonged to SS14-like groups, and a limited number of clinical isolates belonged to Nichols-like groups, which was further verified in our study. Only five Nichols-like strains were identified, accounting for 10.2% of all the studied *T. pallidum* isolates, and the remaining 89.8% were SS14-like strains.

In recent years, there have been several cases of clinical syphilis treatment failure associated with macrolide resistance that was conferred by the A2058G or A2059G mutations in the 23S rRNA (Zhou et al., 2010; Molini et al., 2016). Based on previous molecular analysis, multiple locations in China showed a high prevalence rate (up to over 90%) of macrolide-resistant *T. pallidum* isolates (Martin et al., 2009; Chen et al., 2013; Lu et al., 2015). Similarly, in Xiamen, we detected 38 *T. pallidum* isolates (35 SS14-like isolates and 3 Nichols-like isolates) and found all of them harbored the A2058G mutation, suggesting that macrolide-resistant *T. pallidum* isolates might have spread throughout the country. Recent research using direct whole-genome sequencing combined with phylogenetic analyses revealed that both SS14-like and Nichols-like lineages with genotypic macrolide resistance were simultaneously circulating, which was corroborated by our findings (Beale et al., 2019). Moreover, the study showed that the widespread macrolide-resistant SS14-like isolates in the population were due to macrolide selective pressure rather than expansion of a single fitness advantage resistant SS14-like isolate. In fact, although macrolide antibiotics are an attractive regimen for syphilis treatment, they are not frequently in the list of physicians’ prescriptions for syphilis (Lu et al., 2015). Instead, in China, macrolide antibiotics are widely used in other indications, such as upper respiratory tract infections and other sexually transmitted infections. The use of antibiotics in China was approximately 10-fold higher than that in other nations (Chen et al., 2013). The widespread macrolide-resistant *T. pallidum* isolates may be an indication of broader issues of antimicrobial resistance.

In contrast, all *T. pallidum* isolates in this study had no mutations in the 16S rRNA that could potentially confer tetracycline resistance, consistent with other reports (Xiao et al., 2016; Grillova et al., 2018). In addition, previous studies identified a number of variants affecting penicillin-binding proteins (Sun et al., 2016; Beale et al., 2019). Our study further confirmed the characteristics of homoplastic changes in *tp0760* (I415F/I415M) and *tp0705* (A506V/A506T). Interestingly, variants of *tp0760* were exclusively reported in isolates from China and were not found in isolates from other countries (Beale et al., 2019). Moreover, Mathew et al. identified the variant *tp0705* A506T both in Nichols-like isolates and in SS14-like isolates, but our study showed that the variant *tp0705* A506T only appeared in Nichols-like isolates and that the variant *tp0705* A506V only appeared in SS14-like isolates. The inconsistent results might reflect different evolutionary selection in *T. pallidum* isolates from different geographic areas. However, none of the variants had a significant functional effect on *pbp* genes, according to analysis by SIFT software. Despite this result, we still cannot ignore the possibility of evolutionary pressure causing resistance in an era of uncontrolled antibiotic use, and we need to keep monitoring mutations in *pbp* genes that are putatively involved in a decrease in susceptibility to penicillin (Beale et al., 2019).

Finally, the limitations of this study are discussed. First, the clinical and epidemiological data were not sufficient to further analyze the clinical relevance of the genotypes. Second, the data of syphilis patients regarding the history of antibiotic use or treatment was lacking, and thus, this study could not provide direct evidence about the relationship among treatment failure, macrolide resistance, and genetic mutations in *T. pallidum*.

This study made use of traditional typing scheme (ECDC) and a recently refined typing scheme (MLST) to reveal the genetic diversity of circulating *T. pallidum* isolates in Xiamen between 2016 and 2017. The most common genotype by ECDC was 16d/f and the prevalent allelic profile by MLST was 1.1.8, demonstrating that the group of *T. pallidum* isolates in Xiamen has geographic differences. The findings from this study offer valuable information on syphilis epidemiology as well as syphilis evolution. Moreover, this study for the first time identified genetic mutations associated with antibiotic resistance in Xiamen. It could be a indicator for clinicians in the treatment of syphilis patients.

## Data Availability Statement

The datasets presented in this study can be found in online repositories. The names of the repositories is PubMLST database of Treponema pallidum subsp. pallidum (https://pubmlst.org/tpallidum/).

## Ethics Statement

The study was approved by the Ethics Committee of Zhongshan Hospital, Xiamen University, and complied with the Declaration of Helsinki. All patients provided written consent in accordance with the institutional guidelines.

## Author Contributions

J-JN and T-CY conceived the study. DL, S-MH, and T-CY designed the study. DL, L-RL, and L-LL performed the wet lab experiments. DL and J-JN performed bioinformatics analyses. S-MH and X-ZZ performed research and analyzed data. L-RL performed sample collection. DL, S-MH, and T-CY wrote the manuscript. All authors contributed to the article and approved the submitted version.

## Funding

This work was supported by the National Natural Science Foundation of China [grant numbers 81971147, 81972028, 81973104, 81772260, 81771312, 82001292], the Key Projects for Province Science and Technology Program of Fujian Province [grant number 2018D0014, 2019D008]. The funders played no role in the study design, data collection, or analyses, the decision to publish, or manuscript preparation.

## Conflict of Interest

The authors declare that the research was conducted in the absence of any commercial or financial relationships that could be construed as a potential conflict of interest.
